# Case Reports: Exposure to SARS-CoV-2, Acute Kidney Injury, and Lithium Toxicity

**DOI:** 10.1097/JCP.0000000000001586

**Published:** 2022-08-02

**Authors:** Melvin G. McInnis, Anastasia K. Yocum

**Affiliations:** From the Department of Psychiatry, University of Michigan, Ann Arbor, Michigan.

**Keywords:** bipolar, lithium, SARS-CoV-2, COVID-19

## Abstract

**Purposes:**

The aims of the study were to review 3 cases of lithium toxicity among individuals with bipolar disorder who were diagnosed with COVID-19 and to review the literature discussing the implications of COVID-19 and exposure to SARS-CoV-2 relative to medical use of lithium in management of bipolar disorder.

**Methods:**

This is a case review of medical and psychiatric notes of 3 individuals with bipolar disorder, managed with lithium, who developed COVID-19. This study discussed these cases in context of previous case reports and relevant literature pertaining to lithium and exposure to SARS-CoV-2.

**Findings:**

Infection with SARS-CoV-2 along with symptoms of COVID-19 and mental state changes in three individuals were temporally associated with lithium levels in the toxic range.

**Implications:**

Exposure to SARS-CoV-2 or symptoms suggestive of COVID-19 should result in increased clinical monitoring of individuals taking lithium. Those taking lithium and providers are advised to have a low clinical threshold for requesting lithium levels and kidney function estimates for the duration of the COVD-19 pandemic.

The complications of COVID-19 are wide ranging, affecting most organ systems in a varying and unpredictable manner.^1^ Acute kidney injury quickly emerged as one of the more common complications,^2^ and recent estimates report that nearly one third of hospitalized patients with COVID-19 had biochemical evidence of acute kidney injury.^3^ Early studies indicated that the SARS-CoV-2 virus that causes COVID-19 gained entry into human cells through the angiotension converting enzyme 2 (ACE2) receptors.^4^ The mechanisms by which SARS-CoV-2 directly affects the tubular and parietal cells of the kidney is thought to be through the ACE2-related pathways in the tubular and glomerular systems of the kidney.^5^ Furthermore, tubular injury and glomerular injury are common pathologies found in kidney biopsies of COVID-19 patients.^6^ Additional risk factors include male sex, older age, diabetes, the presence of chronic kidney disease, and hypertension. These findings are highly relevant to clinicians managing individuals taking medications excreted via the kidney. Thus, patients with bipolar disorder taking lithium are of major concern.

Lithium is a simple element with complex biological interactions.^7^ It is the standard of care for maintenance management of bipolar disorder.^8^ The standard of care for therapeutic monitoring of lithium treatment includes regular lithium levels, a metabolic profile with kidney and thyroid function tests.^8^ Once the patient is clinically and biochemically stable, it is recommended that these tests be done at 6-month intervals. However, the risk for lithium toxicity is increased under conditions of metabolic stress that includes dehydration, acute medical illness, and concomitant use of medications affecting the excretion of lithium.^9^ In such circumstances, it is clinically indicated to monitor lithium more frequently.

There are several parallel features of lithium that are of academic and potentially therapeutic relevance. It is, for example, considered to be neurotropic, counteracting oxidative stress and possibly preventing shortening of telomeres associated with the aging process.^10^ Further, a recent study has found that therapeutic levels of lithium are associated with a lower risk of acquiring the illness COVID-19, possibly due to its intrinsic antiviral properties.^11^

While lithium may have protective features that diminish the probability of infection with SARS-CoV-2,^12–14^ many taking lithium still become ill with COVID-19. There have been 3 published case reports of lithium toxicity associated with COVID-19 to date^15–17^ that emphasize the temporal relationship between the illness COVID-19 and lithium toxicity. Herein, we add 2 additional cases that demonstrate the association lithium toxicity with acute kidney injury, as measured by the estimated glomular filtration rate (eGFR) and clinical symptoms. In addition, another case where lithium toxicity emerged in the context of an acute, but mild, COVID-19 illness with lithium toxicity in the immediate wake of the infection.

The importance of close lithium level kidney function monitoring in the context of recent exposure to the SARS-CoV-2 virus is emphasized. It is prudent to advise providers, patients, and families to be aware that lithium levels should be monitored closely whenever there are concerns of SARS-CoV-2 exposure.

## CASE REPORTS

The following 3 case studies emphasize the importance of monitoring lithium levels upon the diagnosis of COVID-19. The individuals identified as patients 1, 2, and 3 reviewed the clinical summaries hereinafter and provided written consent allowing the anonymized clinical information to be shared publicly.

### Patient 1

A 50-year-old married and employed White man with bipolar I disorder had been clinically stable for more than 20 years on lithium 1200 mg daily. He had been receiving community-based medical management for bipolar disorder and insulin-dependent type II diabetes, insulin dependent. He also had a history of peripheral neuropathy and hypertension. Over the past several years, he was managed jointly by his primary care physician and community-based psychiatrist, with minimal concerns or complications. In February 2021, he was diagnosed with COVID-19. The home-managed course of illness of COVID-19 was very mild with a few days of upper respiratory symptoms, no fever, or other symptoms, and he did not lose olfaction. On the day of diagnosis, creatinine was 1.4 mg/dL and his eGFR was 56 mL/min. He was vaccinated mid-March 2021 with second dose (Moderna) 3 weeks later. He was seen by his primary care physician in mid-April 2021 for routine follow-up care for diabetes; no lightheadedness or dizziness was documented; the patient requested an increase in gabapentin for peripheral neuropathy and nighttime peripheral limb discomfort. Two weeks later (end of April 2021), he sought medical care because of nausea, vomiting, and confusion and was admitted urgently to hospital. His medications at the time of admission were lithium 1200 mg daily, metformin 200 mg daily, linisopril 10 mg daily, gabapentin 900 mg daily, citalopram 20 mg daily, atorvastatin 40 mg daily, tadalafil 20 mg as needed, and insulin.

His lithium level on admission was 3.1 mEq/L; eGFR was 20 mL/min and creatinine 3.4 mg/dL. Lithium was immediately discontinued. He received standard care for lithium toxicity including hemodialysis and remained in hospital for 7 days. At the time of discharge, his eGFR was 44 mL/min and creatinine 1.75 mg/dL. Follow-up in September 2021 revealed an eGFR of 61 mL/min and creatinine of 1.35 mg/dL. A review of the pattern of lithium levels documented in the electronic health record: May 2013, 0.7; March 2015, 0.7 mEq/L; May 2019, 0.8; and December 2019, 1.4 mEq/L. There were no data on Li levels in 2020. His eGFR over the past 2 years before the admission in April 2021 ranged between 56 and 61 mL/min; his creatinine was between 1.3 and 1.4 mg/dL.

Given the complexity of his clinical pattern, he was referred to specialty psychiatric care and his bipolar disorder successfully managed with divalproex 1500 mg daily and clonazepam 0.5 mg at bedtime. The option of restarting lithium was discussed and offered; he decided to continue with divalproex as a mood stabilizer. Citalopram and alprazolam were discontinued. He continues in his current employment.

### Patient 2

A 64-year-old retired married White man with bipolar I disorder and chronic kidney disease. He had been treated with lithium at therapeutic levels for more than 25 years with occasional breakthrough manic symptoms managed with antipsychotic medications. Concerns about diabetes insipidus and nocturnal urination prompted discontinuation of lithium in 2012 and management with anticonvulsants and antipsychotic medications, which resulted in several manic episodes and hospitalizations. In 2015, lithium was restarted, which quickly led to mood stabilization but diabetes insipidus returned. He was jointly managed with specialty care in nephrology and psychiatry. Over 2015–2022, his eGFR estimations ranged between 46 and 60 mL/min and creatinine between 1.2 and 1.5 mg/dL. In April 2020, he was directed to the ER for fatigue and confusion. He tested positive for COVID-19. Medications on admission were as follows: amiloride, 5 mg daily; gemfibrozil, 600 mg daily; levothyroxine, 50 μg daily; indomethacin, 75 mg daily; lithium, 600 mg daily; omeprazole, 40 mg daily; and quetiapine, 100 mg daily.

On the day of admission, his Li level was 1.61 mEq/L; creatinine 4.39 mg/dL, and eGFR 20 mL/min. His laboratory results 6 weeks before admission were Li 0.9 mEq/L, creatinine 1.3 mg/dL, and eGFR 58 mL/min. Upon admission, lithium was discontinued and he was managed symptomatically for COVID-19. His hospitalization was 4 weeks due to the severity of COVID-19. The hospital course was complicated by acute kidney injury (lowest eGFR, 9 mL/min), compromised pulmonary function (lowest arterial blood gas, 68), and hypo-hemiglobinemia (lowest value, 5.8). He received supportive management on a specialty medical unit (non-ICU) and gradually recovered. After discharge, he was restarted on lithium and his currently successfully managed on 600 mg daily (0.8 mEq/L) and quetiapine 100 mg daily. He is doing well clinically, most recent (February 2022) eGFR is 60 mL/min and creatinine is 1.3 mg/dL.

### Patient 3

A 56-year-old married White man with bipolar I disorder managed with lithium 1200 mg daily and cariprazine 4.5 mg daily. He has hypothyroidism managed with levothyroxine 150 μg daily, and gastrointestinal reflux successfully managed with omeprazole, 20 mg daily. His bipolar illness was unstable over the past 3 years with ongoing symptoms and nonadherence to treatment. In December 2020, he attempted suicide by CO asphyxiation with subsequent anterograde amnesia. Monitoring of Li levels ranging from 0.7 to 1.0 mEq/L over the past 6 months, electrolytes, eGFR, and thyroid stimulating hormone were unremarkable. His condition and medication were managed with the assistance of a supportive family who monitored administration of all medications. In February 2022, he was diagnosed with COVID-19 and he began experiencing disorientation, confusion, and Parkinson-like symptoms with shuffling gait and masked facies. A lithium level was 1.7 mEq/L, eGFR 78 mL/min, and creatinine 1.08 mg/dL. Lithium and cariprazine were discontinued. His care continued at home, although the option of admission was considered advisable. Within 5 days of discontinuing lithium and cariprazine, his symptoms resolved. He is presently managed with quetiapine 50 mg at bedtime for sleep. Lithium was not restarted at patient and family's request and a plan was implemented to monitor mood variability.

### Discussion

The cases described above highlight the risk of lithium toxicity after exposure to SARS-CoV-2. We emphasize the importance of therapeutic monitoring lithium among individuals taking lithium after any metabolic or medical perturbation, but especially in the current COVID-19 pandemic. In the current (May 2022) environment, home testing for COVID-19 is common, and among those with minimal clinical symptoms, there may be little or no interaction with the health system. However, there are unforeseen complications, and the evidence of the selective binding of the SARS-CoV-2 virus to the ACE2 receptors in the kidney may influence function and compromise lithium excretion. The consequences may be significant. Patient 1, upon experiencing lithium toxicity, elected to not continue on lithium despite the long history of successful management of bipolar disorder with lithium.

In complex clinical circumstances, such as COVID-19, that include metabolic disruption, it is difficult, if not impossible, to assign causality to any one specific feature leading to mental status changes. The common elements in these 3 cases were lithium, COVID-19, and lithium levels in the toxic range. Two of the 3 cases had evidence of acute kidney injury as evidenced by a lowered eGFR. All cases emphasize the importance of ongoing vigilance toward the possibility of dysregulated lithium levels during illness, and these as well as other reports highlight the need for focused attention on the bipolar individual taking lithium who becomes infected with SARS-CoV-2.

The common features of the previous case reports and the current report were the elevated lithium levels and confusion,^15–17^ and all indicators were that lithium had been prescribed appropriately and that the levels had been stable and in a therapeutic range before the acute illness. Patient 1 in our series had an elevated lithium level (1.4 mEq/L) over 1 year before becoming infected with SARS-CoV-2, and his toxic state emerged over 1 month after the infection. The temporal relationship with the SARS-CoV-2 infection could be purely by chance.

Medical and metabolic comorbidities with bipolar disorder, including hypertension and diabetes, are of concern,^18^ and the COVID-19 pandemic only serves to heighten these concerns. A recent report of increased risk of hypernatremia among bipolar patients on long-term lithium during the pandemic further emphasizes the need for sustained vigilance among care providers and awareness among patients and families living with bipolar disorder.^19^

Lithium remains among the most effective long-term management strategies for bipolar disorder.^8^ It has the advantage of being able to monitor blood levels to ensure that the treatment is optimized.^20^ Lithium is excreted via the kidney, and with long-term treatment, renal function must be monitored as lithium treatment can cause compromised function, although with current monitoring strategies and using lower levels, the long-term outcomes are far more favorable.^21^ However, any intervening condition that stands to disrupt the clinical or biochemical equilibrium may adversely affect those with bipolar disorder and especially those treated with lithium. Included among the concerns is the report of atypical antipsychotics and potential risk for acute kidney injury, which would compromise lithium excretion, at least for older people.^22^ Although there are many factors that may contribute to lithium toxicity, the cases described hereinabove in addition to those previously reported, associate lithium toxicity with recent COVID-19 illness and emphasize the importance of monitoring lithium levels immediately after exposure and possibly more frequently in the months after illness.

Patients, providers, and families need to be advised that exposure to the SAR-CoV-2 and related viruses may adversely affect kidney functioning and to request more frequent monitoring of lithium levels. While physical COVID-19 symptoms may be mild and result in minimal respiratory concern, temporary effects on kidney functioning may occur and result in disruption of lithium levels, leaving to toxicity.

## References

[1] AlSamman M Caggiula A Ganguli S, . Non-respiratory presentations of COVID-19, a clinical review. *Am J Emerg Med*. 2020;38:2444–2454. 33039218 10.1016/j.ajem.2020.09.054 PMC7513760

[2] Batlle D Soler MJ Sparks MA, . Acute kidney injury in COVID-19: emerging evidence of a distinct pathophysiology. *J Am Soc Nephrol*. 2020;31:1380–1383. 32366514 10.1681/ASN.2020040419 PMC7350999

[3] Sullivan MK Lees JS Drake TM, . Acute kidney injury in patients hospitalized with COVID-19 from the ISARIC WHO CCP-UK Study: a prospective, multicentre cohort study. *Nephrol Dial Transplant*. 2022;37:271–284. 34661677 10.1093/ndt/gfab303 PMC8788218

[4] Li G He X Zhang L, . Assessing ACE2 expression patterns in lung tissues in the pathogenesis of COVID-19. *J Autoimmun*. 2020;112:102463. 32303424 10.1016/j.jaut.2020.102463 PMC7152872

[5] He Q Mok TN Yun L, . Single-cell RNA sequencing analysis of human kidney reveals the presence of ACE2 receptor: a potential pathway of COVID-19 infection. *Mol Genet Genomic Med*. 2020;8:e1442. 32744436 10.1002/mgg3.1442 PMC7435545

[6] Ferlicot S Jamme M Gaillard F, . The spectrum of kidney biopsies in hospitalized patients with COVID-19, acute kidney injury, and/or proteinuria. *Nephrol Dial Transplant*. 2021;gfab042. doi:10.1093/ndt/gfab042. 33576823 PMC7928708

[7] Haggarty SJ Karmacharya R Perlis RH. Advances toward precision medicine for bipolar disorder: mechanisms & molecules. *Mol Psychiatry*. 2021;26:168–185. 32636474 10.1038/s41380-020-0831-4 PMC10290523

[8] Yatham LN Kennedy SH Parikh SV, . Canadian Network for Mood and Anxiety Treatments (CANMAT) and International Society for Bipolar Disorders (ISBD) 2018 guidelines for the management of patients with bipolar disorder. *Bipolar Disord*. 2018;20:97–170. 29536616 10.1111/bdi.12609 PMC5947163

[9] Groleau G. Lithium toxicity. *Emerg Med Clin North Am*. 1994;12:511–531. 8187694

[10] Salarda EM Zhao NO Lima CNNC, . Mini-review: the anti-aging effects of lithium in bipolar disorder. *Neurosci Lett*. 2021;759:136051. 34139318 10.1016/j.neulet.2021.136051 PMC8324565

[11] De Picker LJ Leboyer M Geddes JR, . Association between serum lithium level and incidence of COVID-19 infection. *Br J Psychiatry*. 2022;221:425–427. doi:10.1192/bjp.2022.42. 35318909 PMC7612897

[12] Murru A Manchia M Hajek T, . Lithium's antiviral effects: a potential drug for COVID-19 disease? *Int J Bipolar Disord*. 2020;8:21. 32435920 10.1186/s40345-020-00191-4 PMC7239605

[13] Soriano-Torres O Noa Romero E González Sosa NL, . Lithium salts as a treatment for COVID-19: pre-clinical outcomes. *Biomed Pharmacother*. 2022;149:112872. 35364381 10.1016/j.biopha.2022.112872 PMC8947939

[14] Liu X Verma A Garcia G Jr., . Targeting the coronavirus nucleocapsid protein through GSK-3 inhibition. *Proc Natl Acad Sci U S A*. 2021;118:e2113401118. 34593624 10.1073/pnas.2113401118 PMC8594528

[15] Suwanwongse K Shabarek N. Lithium toxicity in two coronavirus disease 2019 (COVID-19) patients. *Cureus*. 2020;12:e8384. 32637266 10.7759/cureus.8384 PMC7331926

[16] Danışman Sonkurt M Sonkurt HO. Lithium intoxication in COVID-19: a case report. *Psychiatr Danub*. 2021;33:248–249. 34185757 10.24869/psyd.2021.248

[17] Pai NM Malyam V Murugesan M, . Lithium toxicity at therapeutic doses as a fallout of COVID-19 infection: a case series and possible mechanisms. *Int Clin Psychopharmacol*. 2022;37:25–28. 34686643 10.1097/YIC.0000000000000379 PMC8635074

[18] Salgado ME Sutor B Albright RC Jr., . Every reason to discontinue lithium. *Int J Bipolar Disord*. 2014;2:12. 26092398 10.1186/s40345-014-0012-y PMC4477477

[19] Otani K Mogi K Miura A, . Medical comorbidities leading to hypernatremia during COVID-19 pandemic in patients with mood disorders on long-term lithium therapy. *Bipolar Disord* Advance online publication. 2022. doi:10.1111/bdi.13195. 35181972

[20] Nolen WA Licht RW Young AH, . What is the optimal serum level for lithium in the maintenance treatment of bipolar disorder? A systematic review and recommendations from the ISBD/IGSLI Task Force on treatment with lithium. *Bipolar Disord*. 2019;21:394–409. 31112628 10.1111/bdi.12805 PMC6688930

[21] Nielsen RE Kessing LV Nolen WA, . Lithium and renal impairment: a review on a still hot topic. *Pharmacopsychiatry*. 2018;51:200–205. 29346806 10.1055/s-0043-125393

[22] Hwang YJ Dixon SN Reiss JP, . Atypical antipsychotic drugs and the risk for acute kidney injury and other adverse outcomes in older adults: a population-based cohort study. *Ann Intern Med*. 2014;161:242–248. 25133360 10.7326/M13-2796

